# Regionaler Vergleich spezialisierter ambulanter und (teil-)stationärer schmerzmedizinischer Versorgung in Deutschland

**DOI:** 10.1007/s00482-024-00829-7

**Published:** 2024-09-18

**Authors:** Joachim Erlenwein, Johanna Buchholz, Christoph Weißmann, Beata Hennig, Ursula Marschall, Eberhardt Sumpf, Johannes Nolte, Frank Petzke

**Affiliations:** 1 Klinik für Anästhesiologie, Universitätsmedizin Göttingen, Robert-Koch-Str. 40, 37075 Göttingen, Deutschland; 2 Zentrale Abteilung Physiotherapie, Universitätsmedizin Göttingen, Göttingen, Deutschland; 3 BARMER – Institut für Gesundheitssystemforschung, Berlin, Deutschland; 4 Überörtliche Praxisgemeinschaft für Schmerzmedizin Hann. Münden und Göttingen, Hann. Münden, Deutschland; 5 Pflege- und Pflegefunktionsdienst, Stabsstelle Pflegewissenschaft, Universitätsmedizin Göttingen, Göttingen, Deutschland

**Keywords:** Chronischer Schmerz, Interdisziplinäre multimodale Schmerztherapie, Öffentliche Gesundheitsversorgung, Medizinische Versorgungssicherheit, Erreichbarkeit von Gesundheitsleistungen, Chronic pain, Interdisciplinary multimodal pain therapy, Public health, Health care security or medical care reliability, Accessibility of health services

## Abstract

**Hintergrund, Ziel der Arbeit:**

Chronische Schmerzen erfordern abgestufte Versorgungskonzepte. Ziel dieser Untersuchung ist die regionale Darstellung bzgl. der Erreichbarkeit von speziellen schmerzmedizinischen Versorgungsangeboten aus Patientenperspektive in Deutschland.

**Material und Methoden:**

Für 1000 aus deutschen Postleitzahlen-Ort-Kombinationen randomisiert generierte Modellpatienten wurden mit einem Routenplaner die Fahrzeit mit Auto (IV) und verfügbare Verbindungen im öffentlichen Personenverkehr (ÖPV) zum nächstgelegenen speziellen schmerzmedizinischen ambulanten und (teil-)stationären Versorgungsangebot erhoben.

**Ergebnisse:**

Ambulante Einrichtungen waren je nach Anteil schmerztherapeutischer Versorgung und Vernetzungsstruktur meistens realistisch erreichbar. Universitäre Schmerzambulanzen lagen im IV zu 70 % (80 % ÖPV) in kritischer, zu 49 % (68 % ÖPV) in unrealistischer Erreichbarkeit. Teilstationäre Angebote zur interdisziplinären multimodalen Schmerztherapie (IMST) lagen von 68 % der analysierten Wohnorte im IV (83 % ÖPV) in kritischer und von 49 % (75 % ÖPV) in nicht realistischer Fahrzeitentfernung bei engerer Anbindung bzw. häufigerer Anreise. Stationäre IMST waren häufiger realistisch erreichbar (IV 39 % kritisch, 14 % nicht realistisch, ÖPV 61 % kritisch, 48 % nicht realistisch).

**Diskussion:**

Die Ergebnisse zeigen, dass bundesweit relevante Unterschiede je nach Wohnort in der Erreichbarkeit von Einrichtungen zur speziellen Schmerzbehandlung bestehen. Mit Blick auf die Behandlung eines chronischen Krankheitsbilds mit langfristiger therapeutischer Zielsetzung und mit der Notwendigkeit abgestufter Versorgung offenbaren die Ergebnisse aus Patientenperspektive eine teils kritische Versorgung.

**Zusatzmaterial online:**

Die Online-Version dieses Artikels (10.1007/s00482-024-00829-7) enthält eine Abbildung zu den randomisiert generierten Wohnorten.

## Abstract (en)

**Background and objective:**

Chronic pain requires graduated and staged levels of care. The aim of this study is to provide a regional overview regarding the accessibility of specialized outpatient and (partial) inpatient pain medicine care from the patient’s perspective in Germany.

**Material and methods:**

For 1000 model patients randomly generated from German postal code location combinations, the travelling time by car (individual transport, IT) and available public transport connections (PTC) to the nearest specialized outpatient and inpatient pain medicine clinics and units were assessed using a route planner.

**Results:**

Outpatient facilities (in a practice setting) were mostly realistically accessible depending on the proportion of pain treatment and the networking structure. University pain outpatient clinics were at a critically reachable distance with IT for 70% of the patients (80% with PTC) and had unrealistic accessibility for 49% of the patients with IT (68% with PTC). Interdisciplinary multimodal pain programs in day clinics were at a critically reachable distance for 68% of patients with IT (83% with PTC) and in 49% (75% PTC) at an unrealistic travelling time distance considering the more intense treatment requiring frequent travel. Full inpatient interdisciplinary multimodal treatment was more realistically reachable (IT 39% critical, 14% unrealistic, PTC 61% critical, 48% unrealistic).

**Conclusion:**

The results show relevant nationwide differences in the accessibility of facilities for specialized pain treatment depending on the place of residence. Considering the treatment of a chronic condition with long-term therapeutic goals and the need for graduated care (outpatient and inpatient treatment), the results reveal a partly critical situation from the patient’s perspective.

## Hintergrund und Fragestellung

Chronische Schmerzen betreffen fast ein Drittel der Bevölkerung in Deutschland; zwischen 3 und 7 % haben dabei schmerzbedingt eine hohe Beeinträchtigung der Alltagsfunktion und Lebensqualität [13, 20]. Die jährlichen Behandlungskosten chronischer Schmerzen liegen um die 6–8 % der gesamten Gesundheitsausgaben [16]. Indirekte Gesundheitskosten mit einbezogen, werden die volkswirtschaftlichen Kosten durch chronische Schmerzen in Deutschland pro Jahr auf ca. 40 Mrd. € geschätzt [16, 17]. Vorhandene abgestufte Versorgungsangebote aus ambulanter, teilstationärer und stationärer Interdisziplinärer Multimodaler Schmerztherapie (IMST) sind nicht nur in sich oft heterogen, sondern auch sehr unterschiedlich verfügbar [17, 19, 27, 30]. Unterschiede gewachsener Strukturen resultieren auch aufgrund unterschiedlicher Rahmensetzung der Bundesländer oder Schwerpunktsetzung der Kostenträger auf Landes- oder Regionsebene [1, 2, 10, 17]. Bisher gab es für Deutschland für die stationäre IMST regionale Daten, allerdings mit rein strukturellem Blick auf die Bevölkerungszahl [26]. Daten zur regionalen Verteilung anderer schmerzmedizinischer Angebote fehlten bisher.

Für eine indikationsgerechte und abgestufte Therapie mit zielgerichtetem Ressourceneinsatz steht bei einem erheblich belasteten und in Mobilität und Belastbarkeit erheblich eingeschränkten Patientenkollektiv neben der Frage, welche Behandlungsform geeignet und indiziert ist, jedoch die Frage der Verfügbarkeit bzw. realistischen Erreichbarkeit [9, 11, 35]. Auch mit Blick auf die aktuellen gesundheitspolitischen Reformüberlegungen bedarf es derartiger Kenntnisse für eine zielgerichtete Diskussion der zukünftigen strukturellen Ausgestaltung schmerzmedizinischer Versorgung [26]. Ziel dieser Untersuchung ist die regionale Darstellung bzgl. der Erreichbarkeit von speziellen schmerzmedizinischen Versorgungsangeboten aus Patientenperspektive im Individualverkehr (IV) und dem öffentlichen Personenverkehr (ÖPV) in Deutschland.

## Studiendesign und Untersuchungsmethoden

Zur Darstellung der regionalen Verfügbarkeit ambulanter und (teil-)stationärer schmerzmedizinischer Versorgungsangebote und deren potenzieller Erreichbarkeit aus Patientenperspektive wurden für 1000 fiktive Modellpatienten anhand deutscher Postleitzahlen (PLZ) konkrete Wohnorte randomisiert generiert.

Aufgrund der nicht mit der Bevölkerungsverteilung der Bundesländer korrelierenden Verteilung der verfügbaren PLZ erfolgte zunächst eine Auswahl aus den zum Studienzeitpunkt verfügbaren 12.844 PLZ-Ort-Kombinationen. Dazu wurden die PLZ-Ort-Kombinationen zunächst nach Bundesländern getrennt und dann entsprechend der Verteilung der deutschen Gesamtbevölkerung bundeslandanteilig PLZ-Ort-Kombinationen (= jeweils ein fiktiver „Modellpatient“) aus der Liste der Kombinationen randomisiert ausgewählt (*n* = 1000, siehe Abb. S1 im Online-Zusatzmaterial; [15]).

Zur Charakterisierung der entsprechend zufällig gewonnen Wohnorte wurden folgende Merkmale über das Gemeindeverzeichnis des Statistischen Bundesamts ermittelt [25]:

- Gemeindetyp (große Kreisstadt, kreisangehörige Gemeinde, bewohntes gemeindefreies Gebiet, kreisfreie Stadt, Markt, Stadt, Stadtkreis, Stadtstaat)
- Fläche in km^2^
- Einwohner (gesamt) bzw. Einwohner pro km^2^

Basierend auf der Typologie der Gebietseinheiten (Nomenclature des Unités territoriales statistiques [NUTS]) erfolgte die Zuordnung der Wohnorte zu „urbanen“, „intermediären“ oder „ländlichen“ Regionen. NUTS bezeichnet eine hierarchische Systematik zur eindeutigen Identifizierung und Klassifizierung der räumlichen Bezugseinheiten der amtlichen Statistik in den Mitgliedstaaten der Europäischen Union. Die genutzte Unterkategorie NUTS‑3 („small regions“) entspricht in Deutschland der Gliederung in Landkreise bzw. kreisfreie Städte [3, 23].

## Identifikation ambulanter schmerzmedizinischer Versorgungsangebote in Deutschland

Die Darstellung ambulanter Versorgungsangebote erfolgte anhand universitärer Schmerzambulanzen und der Teilnehmer an der Qualitätssicherungsvereinbarung Schmerztherapie (QSV), inklusiv der teilnehmenden Ermächtigungsambulanzen an den Krankenhäusern. Als analoges Merkmal für eine besonders definierte Versorgungsvoraussetzung in der Schmerzmedizin wurden zuvor „regionale Schmerzzentren“ der Deutschen Gesellschaft für Schmerzmedizin e. V. mit den QSV-Teilnehmern abgeglichen, aber aufgrund der sehr hohen Überschneidung nicht als eigene Gruppe separat analysiert.

Die Identifizierung aller QSV-Teilnehmer erfolgte über die Ziffer EBM 30700 (abgerechnet bei Patienten der BARMER im Zeitraum 2019 bis 2021). Zudem erfolgte die Darstellung über die Ziffern EBM 30702 (QSV-Teilnehmer mit mindestens 50 bis unter 75 % Anteil Schmerztherapie) bzw. EBM 30704 (QSV-Einrichtungen gemäß Anl. 1 der QSV, Therapeuten die „weit überwiegend“ – mindestens 75 % – chron. Schmerzpatienten haben *und* im Rahmen des Genehmigungsverfahrens eine interdisziplinäre Vernetzungsstruktur nachgewiesen haben). Die hier zur Operationalisierung genutzten EBM ermöglichen aufgrund dessen, dass EBM 30700 und 30702 auch von Einrichtungen abgerechnet werden, die EBM 30704 abrechnen können, keine Quantifizierung im Sinne komplett unterschiedlicher Versorger. Dieses Vorgehen ermöglicht lediglich eine unterschiedliche Darstellung mit zunehmendem Anteil an schmerzmedizinischem Schwerpunkt in den Einrichtungen sowie unterschiedlicher Strukturvoraussetzungen.

Universitäre Schmerzambulanzen wurden über die Übersicht anästhesiologischer Ordinariate (ergänzt um Augsburg, noch ohne Bielefeld, da Ordinariat erst ab September 2020) identifiziert (Fokus war zunächst die anästhesiologische Klinik, im zweiten Schritt wurden andere/interdisziplinär getragene Einrichtungen am Standort berücksichtigt; [4, 34]). Es wurden alle Ambulanzen einbezogen, die einen allgemeinen schmerzmedizinischen Fokus hatten, reine Spezialambulanzen (z. B. Kopfschmerzambulanz) wurden nicht berücksichtigt. Einige Universitätskliniken (Berlin, Bochum, München, Witten/Herdecke) haben an mehreren Standorten Schmerzambulanzen, die separat berücksichtigt wurden.

## Identifikation (teil-)stationärer schmerzmedizinischer Versorgungsangebote in Deutschland

Einrichtungen mit stationärer IMST wurden über das Deutsche Krankenhausverzeichnis (DKV) und den OPS 8-918 „Interdisziplinäre multimodale Schmerztherapie“ (Bezugsjahr 2019) identifiziert, wenn diese im Bezugsjahr mindestens vier Mal erbracht wurde. Zur Charakterisierung des Anbieters wurde die Fallzahl für OPS 8-918 (Jahr 2019) erfasst, zudem die Bettenzahl des Krankenhauses, der Träger (öffentlich, privat, freigemeinnützig) und die erbringende Fachabteilung. Wenn zwei Krankenhäuser, die beide im DKV für 2019 separat aufgelistet waren, zum Recherchezeitpunkt fusioniert waren, wurde die höhere Fallzahl des OPS-Codes aus dem Jahr 2019 für die Auswertung übernommen. Wenn die IMST innerhalb eines Verbunds an einen anderen Standort umgezogen war, wurde die Fallzahl aus 2019 übernommen. In der Analyse der Entfernung zur nächsten stationären IMST wurde zunächst die nächste Einrichtung betrachtet, die die OPS 8-918 im Bezugsjahr erbracht hatte, unabhängig von der Fallzahl. Um die Wahrscheinlichkeit fester Routinen, fester Teamstruktur und der Ausrichtung mit Therapieprogramm zu erhöhen, wurde in einem zweiten Schritt nur die nächstgelegene Einrichtung berücksichtigt, die mehr als 75 Fälle/Jahr aufwies. Zwei Kliniken, die unter der OPS 8‑918 ausschließlich Kinder und Jugendliche behandelten, wurden ausgeschlossen.

Einrichtungen mit teilstationärer IMST (= Schmerztagesklinik [TK]) wurden zunächst ebenfalls über das DKV mit dem OPS-Code 8‑91c identifiziert. Einrichtungen, die teilstationär nicht über OPS abrechneten, wurden anhand einer Recherche nach Einrichtungen mit teilstationärem Entgeltschlüssel bei BARMER-Patienten im Zeitraum 2019 bis 2021 ergänzt. Im zweiten Schritt wurde per Recherche verifiziert, welche der hier über Entgeldschlüssel zusätzlich identifizierten Einrichtungen ein tagesklinisches IMST-Therapieprogramm anbieten (Stand Juni 2022). Die Fallzahlen für OPS 8‑91c konnten nur für diejenigen Kliniken dargestellt werden, die im DKV gelistet waren (Stand 2019). Analog zu den stationären Einrichtungen wurden Charakteristika zu Träger und Fachabteilung ermittelt.

## Analyse Anfahrtsweg und Erreichbarkeit

Anhand des Routenplaners Google Maps© (Google Ireland Limited) wurde für jeden der 1000 Modellpatienten die jeweilige Anfahrt nach standardisiertem Protokoll zu festen Zeiten für jede der Versorgungsarten erfasst. Analysiert wurde für jeden der Modellpatienten die jeweilige Entfernung (km), Fahrzeit (min) und Kosten (0,3 €/km) zu der jeweiligen Versorgungsart (ambulante Einrichtungen der Qualitätssicherungsvereinbarung [QSV] identifiziert über die Grundpauschale EBM 30700 und Zusatzpauschale EBM 30702, über die EBM 30704 gesondert dargestellt QSV-Einrichtungen, mit Mindestanteil von 75 % ihrer Leistungen in der Schmerztherapie *und* interdisziplinärer Vernetzungsstruktur; universitäre Schmerzambulanzen; teil- und vollstationäre IMST) mit dem Auto (Individualverkehr, im Folgenden IV) und soweit möglich zu ermitteln die Fahrzeit (min) mit dem öffentlichen Personenverkehr (im Folgenden ÖPV; [4, 34]). Im Kontext der aktuellen gesundheitspolitischen Diskussion um Ambulantisierung, tagesstationäre oder teilstationäre Behandlungen mit Blick auf eine realistische Erreichbarkeit und Umsetzbarkeit, insb. für regelmäßige bzw. tägliche Anfahrten, wurden die Fahrzeiten aus der klinischen Erfahrung der ambulanten und teilstationären interdisziplinären multimodalen Schmerzbehandlung bewertet. Eine Orientierung dabei sind die definierten Grenzwerte für die noch zumutbare Erreichbarkeit eines Facharztes [5, 21]. Hier werden in Abhängigkeit der Facharztzugehörigkeit höchstens 30 min Fahrzeit oder 30 km vom Wohnort angesehen (z. B. für hausärztliche/allgemeinmedizinische Versorgung und allgemeine fachärztliche Versorgung, z. B. Augenärzte, Frauenärzte, Chirurgen, Hautärzte, HNO-Ärzte, Neurologen, Kinder- und Jugendärzte, Orthopäden, Urologen sowie Psychotherapeuten) oder bis zu 60 min Fahrzeit oder 60 km für spezialisierte Fachärzte (z. B. Anästhesiologen, Kinder- und Jugendpsychiater, Fachinternisten [fachärztlich tätig] sowie Radiologen; [5]). Das Bundessozialgericht sieht Wegezeiten als entscheidend an und sieht hier rund 45 min bzw. vereinzelt 60 min als zumutbar an [14].

Unter Berücksichtigung des funktionell eingeschränkten Patientenkollektivs wurden in der vorliegenden Analyse der Erreichbarkeit schmerzmedizinischer Einrichtungen für tägliche Fahrzeiten zu den Versorgungsangeboten für den IV als realistisch Anfahrten von bis zu 30 min bzw. als kritisch bis zu 45 min festgelegt, für den ÖPV realistisch bis 45 min bzw. kritisch bis 60 min.

### Vorgehen zur Recherche des Anfahrtswegs und der Anfahrtszeit

Als Startpunkt für die Analyse „Wohnort bis nächstgelegene Einrichtung“ wurde für den IV das Ergebnis der Suche „Postleitzahl Ort“ im Routenplaner Google Maps© genutzt (diese legt einen Punkt mittig der PLZ fest), wenn innerhalb des ersten Kilometers Wegstrecke eine konkrete Adresse verfügbar war. Da für ÖPV-Analysen eine konkrete Adresse erforderlich ist, wurde die nächste konkrete Adresse inkl. Hausnummer innerhalb des ersten Kilometers Wegstrecke manuell ermittelt und als Ausgangspunkt verwendet. Wenn die Suche „Postleitzahl Ort“ keine konkrete Adresse innerhalb dieser Wegstrecke ergab (= nicht besiedelt), wurde der Startpunkt für die Analyse nur mit Ortsnamen (ohne PLZ) ermittelt und der dann vom Programm festgelegte Ortsmittelpunkt (IV) bzw. die dort nächstgelegene konkrete Adresse (ÖPV) genutzt. Bis 100 km Wegstrecke wurde diese mit Dezimalen angegeben, darüber hinaus gerundet.

Da Google Maps© entsprechend den aktuellen Verkehrsbedingungen Fahrt-Ist-Zeiten angibt, wurden zur Reduktion tageszeitlicher Schwankungen Fahrstrecke und Fahrzeit in festen Zeitfenstern analysiert (montags und dienstags zwischen 8:00 Uhr und 9:30 Uhr). Von den vorgeschlagenen Möglichkeiten wurde jeweils diejenige mit der geringsten Entfernung in km (die ausschließlich auf deutschem Staatsgebiet verlief), bei gleichweiten Routen diejenige mit der kürzeren Fahrzeit genutzt. Lagen auf der Strecke unübliche passagere Verzögerungen (z. B. tageweise Straßensperrung), wurde die Recherche zu einem späteren Zeitpunkt innerhalb des festgelegten Zeitrahmens wiederholt. Für den ÖPV wurde die Fahrzeit ebenfalls soweit möglich mit Google Maps© ermittelt (Funktion „mit öffentlichen Verkehrsmitteln“, Voreinstellung „beste Route“). Es wurden alle öffentlichen Verkehrsmittel inkludiert.

Die von Google Maps© kalkulierte Wegezeit von Tür zu Tür inkludiert Geh- und Wartezeiten. Zur realitätsnahen Abbildung (Annahme Ambulanz- oder Aufnahmetermin am Vormittag) wurden Verbindungen berücksichtigt, die die Ankunft des Modellpatienten am Zielort montags zwischen 8:00 Uhr und 11:00 Uhr ermöglichen. Verbindungsangaben wurden bzgl. Umsetzbarkeit der ÖPV-Verbindung für Patienten nach den folgenden Kriterien gewertet:

- „Gute Verbindung“: kumulierte Laufstrecke ≤ 2 km, ≤ 3 Verkehrsmittel
- „Kritische Verbindung“: kumulierte Laufstrecke > 2 km, > 3 Verkehrsmittel
- „Andere“: keine Verbindung vorhanden oder ermittelbar

Es wurde stets zunächst die zeitlich schnellste ÖPV-Verbindung gewählt. Wenn diese jedoch durch häufigen Wechsel der Verkehrsmittel/lange Gehstrecken (s. oben) als „kritisch“ eingestuft wurde, wurde, wenn verfügbar, die zeitlich nächstlängere Verbindung gewählt, die bzgl. der Kriterien als „gut“ eingestuft wurde und nur maximal 20 % länger dauerte als die „schnellste“ Verbindung. Lag laut Routenplaner nur eine Gehstrecke vor ohne Verfügbarkeit einer schnelleren Verbindung mit ÖPV, wurde diese bis maximal 4 km Gehstrecke für die Anfahrtszeit gewertet, jedoch über 2 km Länge als „schlechte“ Verbindung eingestuft. Für Analysen in Baden-Württemberg, Bayern, Hessen, Mecklenburg-Vorpommern, Rheinland-Pfalz, Saarland, Sachsen und Sachsen-Anhalt waren nur eingeschränkt Daten des ÖPV verfügbar.

### Statistik

Die Analyse erfolgte primär deskriptiv bezogen auf Bundesländer und Wohnortcharakteristika.

## Ergebnisse

### Modellpatienten und Charakterisierung der Wohnorte

Die Wohnorte der 1000 Modellpatienten verteilten sich entsprechenden des Anteils der deutschen Gesamtbevölkerung auf die 16 Bundesländer (siehe Tab. 1). Die Wohnorte in 35 % als urban, in 42 % als intermediär und in 23 % als ländlich typisiert; 49 % waren kreisangehörige Gemeinden, 24 % Städte, 15 % kreisfreie Städte, 7 % Stadtstaaten, 4 % Markt/große Kreisstadt und 1 % andere.

**Tab. 1:** Fahrzeit im Individualverkehr (*IV*) und öffentlichen Personenverkehr (*ÖPV*) nach Wohnortcharakteristika zur jeweils nächstgelegenen schmerzmedizinischen Versorgungseinrichtung

Bundesland		EBM 30700		EBM 30702		EBM 30704		Universitäre Schmerzambulanz		Teilstationäre IMST		Stationäre IMST	
		*Fahrzeit IV*	*Fahrzeit ÖPV*	*Fahrzeit IV*	*Fahrzeit ÖPV*	*Fahrzeit IV*	*Fahrzeit ÖPV*	*Fahrzeit IV*	*Fahrzeit ÖPV*	*Fahrzeit IV*	*Fahrzeit ÖPV*	*Fahrzeit IV*	*Fahrzeit ÖPV*
		*MW* *±* *SD*	*MW* *±* *SD*	*MW* *±* *SD*	*MW* *±* *SD*	*MW* *±* *SD*	*MW* *±* *SD*	*MW* *±* *SD*	*MW* *±* *SD*	*MW* *±* *SD*	*MW* *±* *SD*	*MW* *±* *SD*	*MW* *±* *SD*
		*[min.]*	*[min.]*	*[min.]*	*[min.]*	*[min.]*	*[min.]*	*[min.]*	*[min.]*	*[min.]*	*[min.]*	*[min.]*	*[min.]*
Deutschland *n* = 1000	Urban	11,6 ± 7,0	27,7 ± 22,3	11,8 ± 7,3	28,2 ± 22,6	14,2 ± 8,2	32,0 ± 23,6	28,9 ± 15,7	59,4 ± 32,3	43,5 ± 25,8	84,9 ± 45,0	20,8 ± 12,0	45,5 ± 29,2
	Intermediär	17,7 ± 9,0	45,2 ± 30,2	17,9 ± 9,2	46,3 ± 31,2	22,6 ± 11,8	55,1 ± 33,3	52,3 ± 21,5	103,8 ± 41,4	52,6 ± 30,2	109,2 ± 55,6	30,8 ± 16,4	81,5 ± 45,2
	Ländlich	23,1 ± 11,9	59,9 ± 38,8	23,2 ± 12,0	59,1 ± 38,2	33,5 ± 17,5	81,6 ± 43,9	63,8 ± 23,3	123,1 ± 46,1	50,7 ± 30,0	113,1 ± 55,5	38,6 ± 23,7	91,5 ± 42,2
Baden-Württemberg (BW) *n* = 134	Urban	14,1 ± 6,7	31,9 ± 16,6	14,4 ± 6,9	32,9 ± 16,6	16,9 ± 8,3	35,3 ± 16,1	55,2 ± 19,9	101,5 ± 29,8	70,7 ± 22,2	102,7 ± 29,4	29,0 ± 9,3	66,1 ± 28,5
	Intermediär	17,9 ± 9,1	51,3 ± 33,5	17,9 ± 9,1	52,0 ± 33,5	23,8 ± 11,5	59,4 ± 32,1	56,2 ± 24,4	99,0 ± 44,3	63,3 ± 27,8	107,3 ± 43,7	37,9 ± 21,6	94,3 ± 58,9
	Ländlich	21,2 ± 6,2	58,4 ± 36,1	21,2 ± 6,2	58,4 ± 36,1	23,8 ± 8,3	59,5 ± 36,7	68,9 ± 19,4	144,2 ± 56,2	74,6 ± 25,1	124,2 ± 42,1	30,7 ± 9,9	78,1 ± 28,3
Bayern (BY) *n* = 158	Urban	15,1 ± 10,1	31,5 ± 27,2	15,1 ± 10,1	31,5 ± 27,2	16,8 ± 10,0	30,9 ± 20,1	23,5 ± 7,8	49,2 ± 28,4	21,5 ± 14,1	48,5 ± 32,8	29,0 ± 15,7	65,6 ± 38,5
	Intermediär	16,2 ± 7,8	38,7 ± 28,5	16,2 ± 7,8	39,2 ± 28,5	22,4 ± 11,5	52,5 ± 49,4	44,9 ± 18,3	85,9 ± 44,0	25,4 ± 12,0	60,9 ± 26,4	31,2 ± 15,3	90,2 ± 44,4
	Ländlich	23,2 ± 12,1	59,8 ± 37,5	23,1 ± 12,1	59,8 ± 37,5	33,4 ± 11,4	84,5 ± 34,8	57,2 ± 18,7	96,5 ± 27,3	29,9 ± 13,1	78,2 ± 30,2	36,6 ± 16,2	85,5 ± 36,5
Berlin (BE) *n* = 44	Urban	7,7 ± 4,3	16,9 ± 10,1	7,7 ± 4,4	16,9 ± 10,1	13,7 ± 7,1	24,5 ± 13,2	20,8 ± 9,8	35,1 ± 17,0	18,2 ± 6,8	37,2 ± 13,2	15,6 ± 7,8	30,7 ± 14,3
	Intermediär	–	–	–	–	–	–	–	–	–	–	–	–
	Ländlich	–	–	–	–	–	–	–	–	–	–	–	–
Brandenburg (BB) *n* = 30	Urban	–	–	–	–	–	–	–	–	–	–	–	–
	Intermediär	17,3 ± 8,4	41,2 ± 38,8	18,0 ± 9,5	45,3 ± 41,2	31,5 ± 18,0	51,1 ± 26,7	60,4 ± 22,9	96,2 ± 31,5	40,2 ± 18,1	74,3 ± 28,1	27,7 ± 14,9	66,2 ± 43,2
	Ländlich	23,0 ± 8,5	50,9 ± 26,3	23,0 ± 8,5	50,9 ± 26,3	46,0 ± 21,7	110,4 ± 46,1	77,0 ± 23,7	145,6 ± 32,4	47,2 ± 10,3	104,6 ± 39,5	40,7 ± 17,8	104,4 ± 34,7
Bremen (HB) *n* =8	Urban	10,1 ± 6,7	19,4 ± 11,5	10,1 ± 6,7	19,4 ± 11,5	10,1 ± 7,1	20,3 ± 12,0	43,9 ± 8,8	80,1 ± 11,7	84,0 ± 8,5	104,9 ± 18,5	18,0 ± 15,2	22,7 ± 8,7
	Intermediär	–	–	–	–	–	–	–	–	–	–	–	–
	Ländlich	–	–	–	–	–	–	–	–	–	–	–	–
Hamburg (HH) *n* = 22	Urban	9,6 ± 5,5	21,9 ± 14,8	9,6 ± 5,5	21,9 ± 14,8	9,9 ± 5,6	22,2 ± 15,2	24,9 ± 10,6	50,5 ± 22,8	15,6 ± 8,4	33,2 ± 19,4	17,1 ± 8,3	33,7 ± 16,1
	Intermediär	–	–	–	–	–	–	–	–	–	–	–	–
	Ländlich	–	–	–	–	–	–	–	–	–	–	–	–
Hessen (HE) *n* = 76	Urban	19,8 ± 9,7	52,0 ± 28,4	19,8 ± 9,7	52,0 ± 28,4	21,3 ± 11,5	56,9 ± 30,7	32,7 ± 11,0	67,7 ± 30,2	44,5 ± 13,4	81,5 ± 33,1	31,1 ± 16,6	61,2 ± 33,4
	Intermediär	16,4 ± 7,7	41,3 ± 25,0	16,4 ± 7,7	41,3 ± 25,0	16,9 ± 7,7	42,5 ± 25,3	41,7 ± 17,4	93,2 ± 40,2	47,9 ± 22,1	120,5 ± 54,2	26,5 ± 10,5	80,6 ± 41,3
	Ländlich	21,7 ± 8,9	62,7 ± 43,7	21,7 ± 8,9	62,7 ± 43,7	31,4 ± 12,5	95,5 ± 53,2	60,3 ± 17,9	119,8 ± 58,6	56,4 ± 14,0	106,7 ± 37,2	39,0 ± 14,0	95,2 ± 36,4
Mecklenburg-Vorpommern (MV) *n* = 19	Urban	–	–	–	–	–	–	–	–	–	–	–	–
	Intermediär	20,7 ± 6,5	66,3 ± 53,7	20,7 ± 6,5	66,3 ± 53,7	28,7 ± 11,8	67,0 ± 36,0	31,0 ± 8,2	95,0–50,5	26,3 ± 13,8	90,0 ± 51,8	78,7 ± 18,2	203,0 ± 18,4
	Ländlich	22,8 ± 10,1	90,4 ± 83,4	22,8 ± 10,1	90,4 ± 83,4	53,4 ± 25,7	109,6 ± 75,3	55,7 ± 25,7	105,1 ± 37,8	37,4 ± 20,4	95,1 ± 86,2	80,0 ± 45,9	173,9 ± 58,3
Niedersachsen (NI) *n* = 96	Urban	11,0 ± 3,6	23,3 ± 17,2	11,0 ± 3,6	23,3 ± 17,2	11,0 ± 3,6	23,3 ± 17,2	12,3 ± 3,8	31,0 ± 11,0	110,0 ± 22,6	144,3 ± 52,3	12,0 ± 3,6	30,7 ± 15,5
	Intermediär	20,8 ± 9,7	53,6 ± 30,4	21,4 ± 9,9	55,5 ± 32,1	25,0 ± 10,7	59,8 ± 32,0	58,0 ± 20,1	111,5 ± 34,9	82,0 ± 33,3	138,6 ± 57,8	31,0 ± 14,9	87,1 ± 50,0
	Ländlich	26,7 ± 9,4	65,3 ± 31,4	27,7 ± 10,4	61,2 ± 27,7	33,3 ± 13,2	75,6 ± 37,1	70,1 ± 22,0	137,8 ± 42,6	81,7 ± 25,8	155,9 ± 55,9	30,0 ± 12,6	78,2 ± 32,2
Nordrhein-Westfalen (NW) *n* = 216	Urban	10,9 ± 5,8	26,9 ± 23,2	11,2 ± 6,4	27,8 ± 23,9	13,0 ± 7,4	31,6 ± 25,8	26,0 ± 12,5	57,1 ± 28,5	50,7 ± 21,0	106,2 ± 40,0	18,3 ± 9,5	43,6 ± 29,0
	Intermediär	18,7 ± 10,1	44,4 ± 30,5	19,5 ± 10,6	46,9 ± 33,0	21,7 ± 13,3	50,4 ± 31,0	49,3 ± 17,3	105,6 ± 33,7	60,4 ± 25,2	136,5 ± 56,8	29,2 ± 12,3	77,8 ± 34,1
	Ländlich	–	–	–	–	–	–	–	–	–	–	–	–
Rheinland-Pfalz (RP) *n* = 49	Urban	10,8 ± 3,6	39,0 ± 14,5	10,8 ± 3,6	39,0 ± 14,5	13,5 ± 4,8	50,7 ± 9,7	34,3 ± 6,8	85,0 ± 29,4	18,3 ± 10,4	64,3 ± 32,0	12,0 ± 5,7	48,5 ± 11,4
	Intermediär	17,4 ± 7,7	58,4 ± 52,8	17,4 ± 7,7	58,4 ± 52,8	21,1 ± 11,1	74,1 ± 56,7	50,4 ± 14,1	83,3 ± 35,1	26,2 ± 10,7	45,8 ± 42,3	28,1 ± 14,6	75,7 ± 47,7
	Ländlich	22,7 ± 8,8	59,8 ± 46,9	22,8 ± 8,8	59,8 ± 46,9	24,2 ± 9,7	59,8 ± 46,9	62,8 ± 21,1	95,5 ± 58,7	34,5 ± 13,9	63,0 ± 29,1	36,1 ± 16,3	80,5 ± 33,9
Saarland (SL) *n* = 12	Urban	11,3 ± 5,9	21,7 ± 9,4	11,3 ± 5,9	21,7 ± 9,4	11,3 ± 5,9	21,7 ± 9,4	33,1 ± 11,8	81,0 ± 36,2	34,3 ± 8,2	84,0 ± 26,7	22,7 ± 7,7	53,3 ± 21,1
	Intermediär	7,7 ± 5,0	17,0 ± 7,5	7,7 ± 5,0	17,0 ± 7,5	7,7 ± 5,0	17,0 ± 7,5	37,0 ± 16,6	90,3 ± 17,8	37,7 ± 17,6	87,3 ± 15,7	28,7 ± 20,5	59,3 ± 25,6
	Ländlich	–	–	–	–	–	–	–	–	–	–	–	–
Sachsen (SN) *n* = 49	Urban	11,6 ± 8,2	33,6 ± 27,9	11,6 ± 8,2	33,6 ± 27,9	18,7 ± 8,6	42,6 ± 25,1	26,1 ± 19,2	56,5 ± 45,5	24,9 ± 17,2	55,3 ± 43,7	27,7 ± 20,1	41,5 ± 31,5
	Intermediär	15,0 ± 9,5	36,4 ± 20,0	15,0 ± 9,5	36,4 ± 20,0	18,6 ± 10,8	42,6 ± 21,8	77,7 ± 15,8	146,8 ± 47,8	39,2 ± 23,0	75,4 ± 28,6	23,2 ± 12,9	62,3 ± 32,7
	Ländlich	22,3 ± 11,4	57,2 ± 34,6	22,3 ± 11,4	57,2 ± 34,6	38,4 ± 15,0	78,1 ± 40,6	57,4 ± 19,1	81,7 ± 21,1	41,9 ± 18,7	78,3 ± 30,7	40,0 ± 14,1	81,7 ± 29,4
Sachsen-Anhalt (ST) *n* = 26	Urban	–	–	–	–	–	–	–	–	–	–	–	–
	Intermediär	17,1 ± 8,2	36,5 ± 18,0	17,0 ± 8,1	36,5 ± 18,0	21,1 ± 8,3	51,5 ± 20,0	39,5 ± 16,4	87,8 ± 27,4	57,2 ± 19,8	110,2 ± 31,1	23,8 ± 9,4	69,6 ± 27,7
	Ländlich	17,9 ± 7,5	27,0 ± 9,6	17,9 ± 7,5	27,0 ± 9,6	36,6 ± 14,6	47,3 ± 36,2	64,4 ± 17,2	104,8 ± 27,9	63,8 ± 11,0	95,5 ± 19,8	28,9 ± 16,9	50,7 ± 35,1
Schleswig-Holstein (SH) *n* = 35	Urban	13,8 ± 5,9	35,0 ± 10,6	13,8 ± 5,9	35,0 ± 10,6	14,8 ± 6,8	38,5 ± 20,1	27,8 ± 10,2	61,5 ± 17,0	53,3 ± 26,6	111,5 ± 63,2	22,5 ± 5,8	58,8 ± 21,4
	Intermediär	18,9 ± 9,0	43,1 ± 24,8	18,9 ± 9,0	43,1 ± 24,8	20,8 ± 8,7	44,8 ± 23,2	40,5 ± 16,5	85,8 ± 28,9	52,6 ± 27,4	113,1 ± 61,1	30,0 ± 9,2	74,1 ± 24,9
	Ländlich	30,5 ± 33,0	51,6 ± 18,7	30,5 ± 33,0	51,6 ± 18,7	42,8 ± 43,7	91,7 ± 41,3	86,5 ± 45,8	164,5 ± 22,7	105,2 ± 54,7	201,8 ± 31,7	43,1 ± 49,2	82,1 ± 35,3
Thüringen (TH) *n* = 26	Urban	–	–	–	–	–	–	–	–	–	–	–	–
	Intermediär	16,3 ± 7,7	40,8 ± 21,1	16,3 ± 7,7	40,8 ± 21,1	25,6 ± 12,7	62,6 ± 35,7	50,9 ± 19,9	126,8 ± 47,8	43,6 ± 17,5	115,5 ± 38,8	27,7 ± 18,2	78,2 ± 55,8
	Ländlich	20,7 ± 9,7	53,7 ± 40,3	20,7 ± 9,7	53,7 ± 40,3	28,8 ± 13,8	78,6 ± 47,7	70,2 ± 29,8	148,2 ± 53,8	56,3 ± 20,1	121,0 ± 39,7	35,8 ± 10,6	111,6 ± 40,7

Die Wohnorte hatten im Minimum eine Fläche von 1,29 km^2^ bis maximal 891,12 km^2^, im Durchschnitt 124,35 km^2^ ± 208,78 km^2^ (Median 45,73 [20,46–105,15]). Die Spannweite der Einwohnerzahl der Wohnorte umfasste zum Analysezeitpunkt 84 bis 3.669.491 Einwohner, durchschnittlich 285.508,76 ± 798.342,28 (793,8 ± 1114,82/km^2^, min. 8, max. 4777/km^2^).

### Ambulante Versorgung

Über die Abrechnung EBM 30700 wurden bundesweit 1089 Standorte als QSV-Einrichtungen (nicht Einzeltherapeuten!) und über EBM 30702 1067 Einrichtungen identifiziert. Über EBM 30704 wurden bundesweit 525 Einrichtungen identifiziert, die einen Mindestanteil von 75 % ihrer Leistungen in der Schmerztherapie hatten und eine interdisziplinäre Vernetzungsstruktur nachgewiesen haben mussten (EBM 30704).

**Abb. 1:**
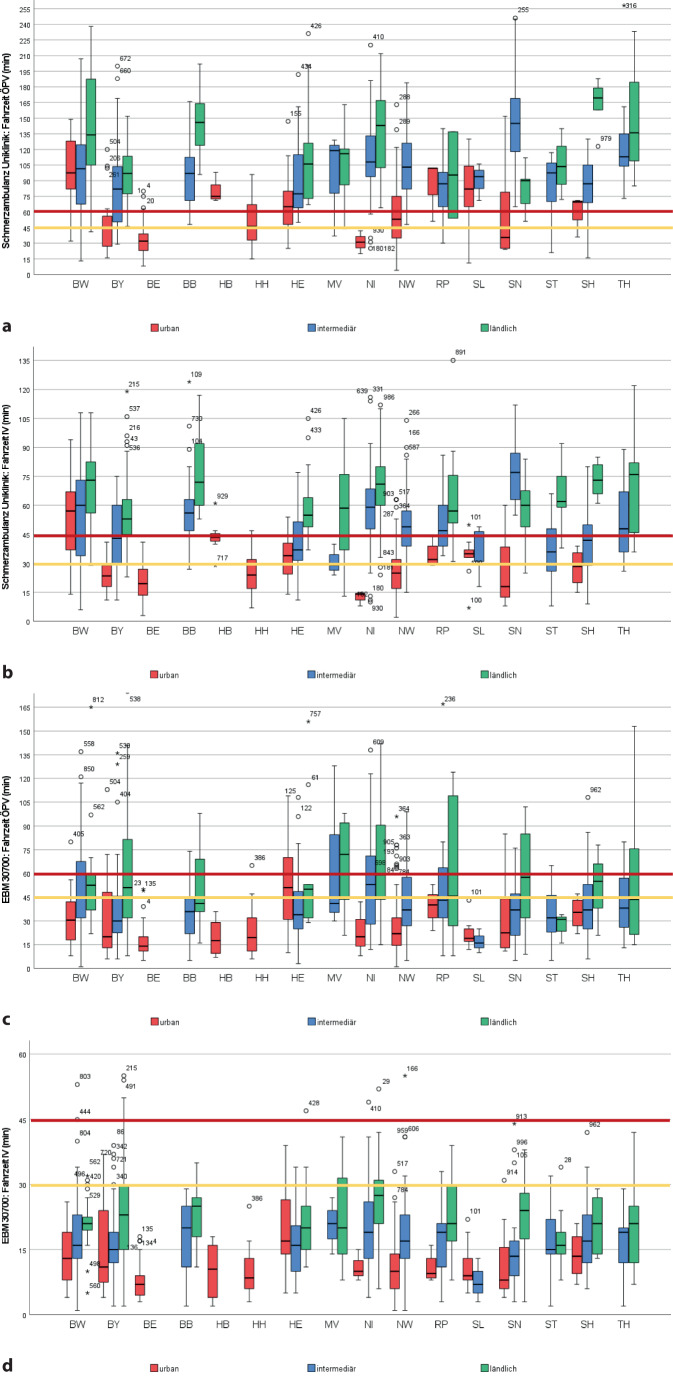
Grafische Darstellung der Erreichbarkeit in Abhängigkeit von Bundesland und NUTS-Region der jeweils nächsten universitären Schmerzambulanz und Einrichtung mit Teilnahme an der Qualitätssicherungsvereinbarung (*QSV*), (Abbrechung EBM 30700) (Universitäre Schmerzambulanz: **a** öffentlicher Personenverkehr [*ÖPV*], **b** Individualverkehr [*IV*]; QSV-Einrichtung EBM 30700: **c** ÖPV, **d** IV)

Eine QSV-Einrichtung unabhängig vom Anteil Schmerztherapie (EBM 30700 und EBM 30702) lag mit durchschnittlich 12,94 ± 10,09 bzw. 13,11 ± 10,21 km meist im näheren Umfeld des Wohnorts (Tab. 1) mit rechnerisch daraus resultierenden Fahrtkosten (nur IV) pro Strecke von durchschnittlich 3,89 ± 3,03 € (min. 0, max. 17,88 €). Für alle Modellpatienten lag eine solche Einrichtung bis maximal 60 km entfernt vor und war sowohl im IV (bis 99 %) als auch ÖPV (bis 81 %) bzgl. der Fahrzeit realistisch zu erreichen (Tab. 1 und 2 sowie Abb. 1 und 2).

**Tab. 2:** Erreichbarkeit der jeweils nächstgelegenen ambulanten Versorgungseinrichtung mit Teilnahme an der Qualitätssicherungsvereinbarung (*QSV*), dargestellt für Individualverkehr (IV) und öffentlichen Personenverkehr (*ÖPV*)

Bundesland	EBM 30700									EBM 30702									EBM 30704								
	*Fahrzeit IV*				*Fahrzeit ÖPV*					*Fahrzeit IV*				*Fahrzeit ÖPV*					*Fahrzeit IV*				*Fahrzeit ÖPV*				
	*Cut-off* *30* *min*		*Cut-off* *45* *min*		*Cut-off* *45* *min*		*Cut-off* *60* *min*		*Anteil ohne Verbindung*	*Cut-off* *30* *min*		*Cut-off* *45* *min*		*Cut-off* *45* *min*		*Cut-off* *60* *min*		*Anteil ohne Verbindung*	*Cut-off* *30* *min*		*Cut-off* *45* *min*		*Cut-off* *45* *min*		*Cut-off* *60* *min*		*Anteil ohne Verbindung*
	*% der verfügbaren Verbindungen*				*% der verfügbaren Verbindungen*				*% von Gesamtzahl*	*% der verfügbaren Verbindungen*				*% der verfügbaren Verbindungen*				*% von Gesamtzahl*	*% der verfügbaren Verbindungen*				*% der verfügbaren Verbindungen*				*% von Gesamtzahl*
	*≤* *30*	*>* *30*	*≤* *45*	*>* *45*	*≤* *45*	*>* *45*	*≤* *60*	*>* *60*		*≤* *30*	*>* *30*	*≤* *45*	*>* *45*	*≤* *45*	*>* *45*	*≤* *60*	*>* *60*	*–*	*≤* *30*	*>* *30*	*≤* *45*	*>* *45*	*≤* *45*	*>* *45*	*≤* *60*	*>* *60*	*–*
Deutschland *n* = 1000	91	9	99	1	68	32	81	19	18	90	10	99	1	67	33	81	19	18	76	24	94	6	57	43	73	27	18
Baden-Württemberg (BW) *n* = 134	94	6	99	1	60	40	80	20	32	94	6	99	1	60	40	80	20	30	77	23	98	2	49	51	73	27	30
Bayern (BY) *n* = 158	85	15	98	2	62	38	79	21	39	85	15	97	3	62	37	79	21	39	64	36	91	10	48	52	69	31	39
Berlin (BE) *n* = 44	100	0	100	0	95	5	100	0	0	100	0	100	0	95	5	100	0	0	98	2	100	0	89	11	98	2	0
Brandenburg (BB) *n* = 30	93	7	100	0	70	30	83	17	0	90	10	100	0	67	33	80	20	0	40	60	77	23	40	60	53	47	0
Bremen (HB) *n* = 8	100	0	100	0	100	0	100	0	0	100	0	100	0	100	0	100	0	0	100	0	100	0	100	0	100	0	0
Hamburg (HH) *n* = 22	100	0	100	0	91	9	95	5	0	100	0	100	0	91	9	95	5	0	100	0	100	0	91	9	95	5	0
Hessen (HE) *n* = 76	88	12	99	1	55	45	73	27	18	88	12	99	1	55	45	73	27	18	78	22	95	5	51	49	64	36	18
Mecklenburg-Vorpommern (MV) *n* = 19	74	26	100	0	40	60	50	50	47	74	26	100	0	40	60	50	50	47	26	74	53	47	20	80	20	80	47
Niedersachsen (NI) *n* = 96	79	21	98	2	43	57	62	38	2	76	24	98	2	41	59	62	38	2	59	41	92	8	35	65	53	47	2
Nordrhein-Westfalen (NW) *n* = 216	97	3	99	1	82	18	90	10	1	96	4	99	1	79	21	89	11	1	94	7	99	1	74	26	86	14	1
Rheinland-Pfalz (RP) *n* = 49	88	12	100	0	56	44	75	25	67	88	12	100	0	56	44	75	25	67	80	20	98	2	44	56	69	31	67
Saarland (SL) *n* = 12	100	0	100	0	100	0	100	0	0	100	0	100	0	100	0	100	0	0	100	0	100	0	100	0	100	0	0
Sachsen (SN) *n* = 49	90	10	100	0	68	32	78	22	16	90	10	100	0	68	32	78	22	16	76	25	94	6	54	47	74	26	16
Sachsen-Anhalt (ST) *n* = 26	92	8	100	0	69	31	87	13	38	92	8	100	0	69	31	87	13	38	69	31	85	15	50	50	57	43	38
Schleswig-Holstein (SH) *n* = 35	91	9	97	3	62	38	79	21	3	91	9	97	3	62	38	79	21	3	80	20	97	3	50	50	65	35	3
Thüringen (TH) *n* = 26	96	4	100	0	56	44	76	24	4	96	4	100	0	56	44	76	24	4	73	26,9	89	12	24	76	56	44	4

**Abb. 2:**
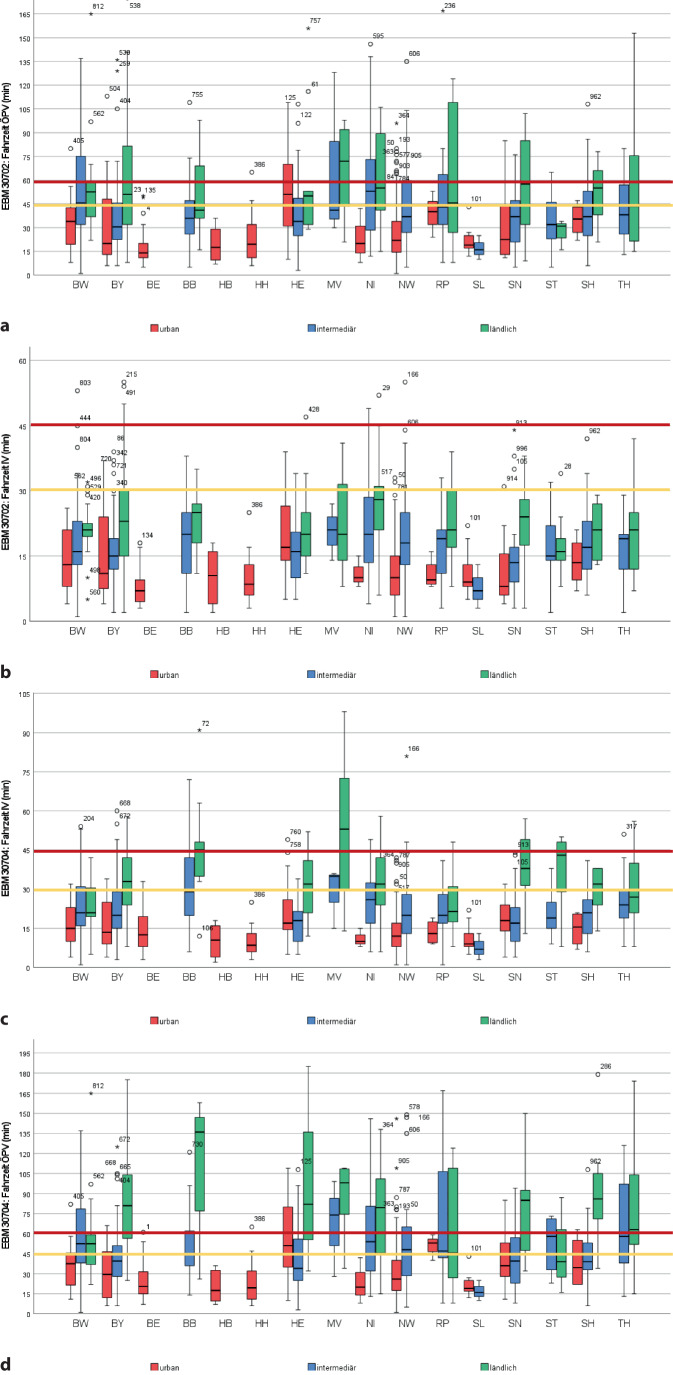
Grafische Darstellung der Erreichbarkeit in Abhängigkeit von Bundesland und NUTS-Region der jeweils nächsten Einrichtung mit Teilnahme an der Qualitätssicherungsvereinbarung (*QSV*) mit mindestens 50 bis unter 75 % Anteil Schmerztherapie (Abrechnung EBM 30702) sowie der QSV-Einrichtung gemäß Anl. 1 der QSV, die „weit überwiegend“/mindestens 75 % chronische Schmerzpatienten behandeln *und* im Rahmen des Genehmigungsverfahrens eine interdisziplinäre Vernetzungsstruktur nachgewiesen haben (Abrechnung EBM 30704) (QSV-Einrichtung EBM 30702: **a** öffentlicher Personenverkehr [*ÖPV*], **b** Individualverkehr [*IV*]; QSV-Einrichtung EBM 30704: **c** ÖPV, **d** IV)

Die nächstgelegene QSV-Einrichtung, in der „weit überwiegend“ (EBM 30704) chronische Schmerzpatienten betreut wurden, lag mit durchschnittlich 18,41 ± 15,97 km (bis max. 117 km) weiter entfernt. Fahrtkosten (nur IV) lagen pro Strecke demensprechend durchschnittlich von 5,52 ± 4,79 € vor (min. 0, max. 35,10 €). Für 19 % der Patienten lag zum Erreichen der nächsten Einrichtung keine ÖPV-Verbindung vor. In 90 % der Fälle wurde die vorhandene ÖPV-Verbindung als „gut“ eingestuft, in 10 % als schlecht. Wenn auch für viele Patienten die Erreichbarkeit einer ambulanten Schwerpunkteinrichtung innerhalb einer realistischen Fahrzeit lag, gab es je nach Bundesland- und Regionstypologie relevante Anteile an Wohnorten, von denen die Erreichbarkeit außerhalb eines realistischen Zeitfensters lag (Tab. 2; Abb. 1 und 2).

### Universitäre Schmerzambulanzen

Es wurden insgesamt 46 universitäre Standorte mit Schmerzambulanzen einbezogen, die durchschnittlich von den Wohnorten 47,47 ± 31,08 km (min. 0,5, max. 161 km, Tab. 1) entfernt lagen. Dies entsprach pro Strecke Fahrtkosten von 14,21 ± 9,33 € (min. 0, max. 48,3 €). Diese Schmerzambulanzen waren bzgl. Fahrzeit für 51 % (IV) bzw. 32 % (ÖPV) der Modellpatienten realistisch erreichbar. Im ÖPV wurden 78 % der Verbindungen als gut eingestuft, 22 % als schlecht und für fast ein Viertel der Patienten war keine Verbindung verfügbar (*n* = 242). Auch hier zeigten sich für Menschen in ländlichen Regionen, teils deutlich, längere Anfahrtswege. Insgesamt liegt für relevante Anteile der Bevölkerung die Erreichbarkeit einer universitären Schmerzambulanz außerhalb der bei einer engeren therapeutischen Anbindung realistisch bzw. kritisch einzuschätzenden Fahrzeit (Tab. 1; Abb. 1).

### Teilstationäre IMST

Es wurden 93 Schmerztageskliniken identifiziert. Die Träger waren mehrheitlich öffentlich (59 %, freigemeinnützig 22 %, privat 19 %). Die häufigsten Organisationsformen waren eine eigenständige Klinik/Abteilung (46 %) und die Zugehörigkeit zur anästhesiologischen Klinik (45 %, 9 % sonstige: je *n* = 3 Orthopädie-Unfallchirurgie und Neurologie, *n* = 2 Psychosomatik). Die Leitung erfolgte meist durch einen Facharzt für Anästhesiologie (80 %, 8 % Neurologie, 12 % sonstige: *n* = 2 je Innere Medizin, Neurochirurgie, Orthopädie-Unfallchirurgie, physikalische Medizin, *n* = 1 je Rehabilitationsmedizin, Psychosomatik, *n* = 7 nicht ermittelbar).

**Abb. 3:**
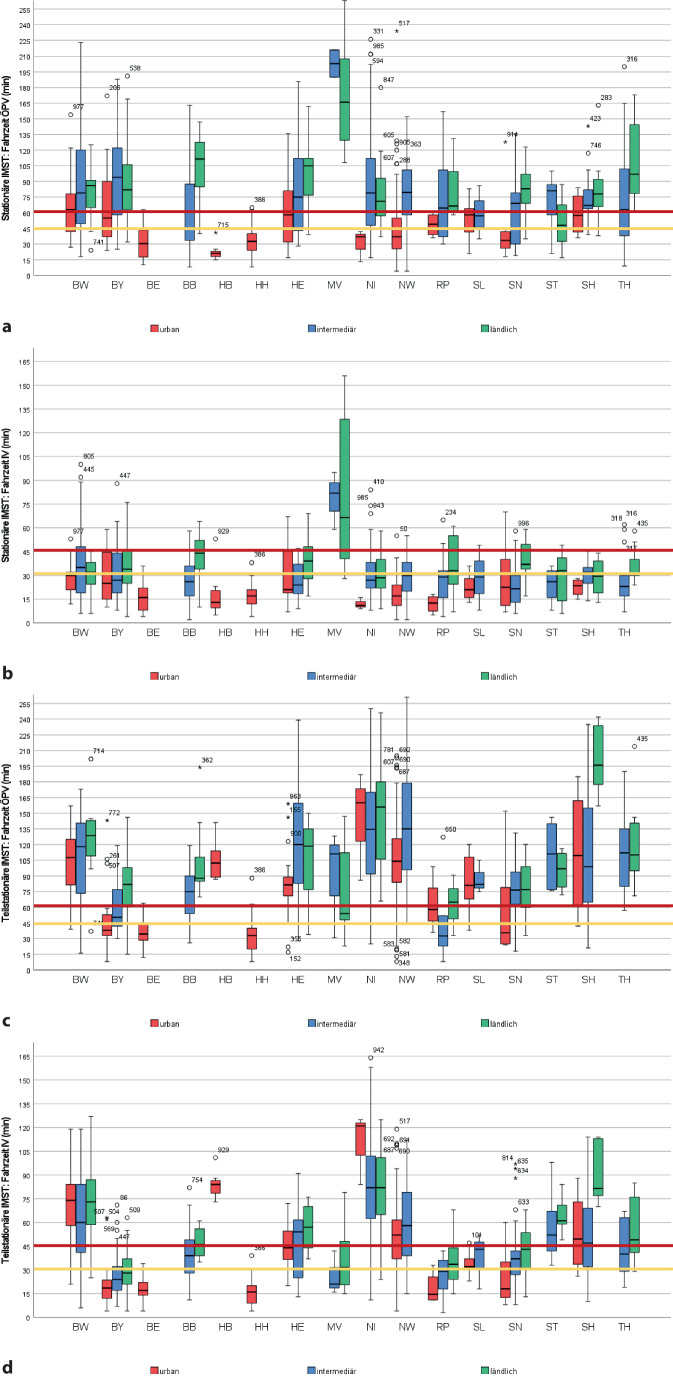
Grafische Darstellung der Erreichbarkeit in Abhängigkeit von Bundesland und NUTS-Region der jeweils nächsten Einrichtung zur (teil-)stationären Interdisziplinären Multimodalen Schmerztherapie (*IMST*) (vollstationäre IMST mit mind. 75 Behandlungsfällen OPS 8–918/Jahr [Bezugsjahr 2019]: **a** öffentlicher Personenverkehr [*ÖPV*], **b** Individualverkehr [*IV*]; teilstationäre IMST: **c** ÖPV, **d** IV)

Durchschnittlich war die nächstgelegene TK 50,82 ± 37,13 km (min. 0,8, max. 237 km) vom Wohnort entfernt. Bzgl. Fahrtkosten entsprach dies durchschnittlich 15,25 ± 11,14 €/einfache Strecke (min. 0,24, max. 71,10 €). Die Fahrzeit im ÖPV zur nächstgelegenen TK betrug im Mittel 98,91 ± 52,58 min/einfache Strecke (min. 8, max. 308 min, Tab. 1). Die Fahrzeit war bzgl. der Erreichbarkeit für 68 % aller Patienten mit dem Auto und für 83 % aller Patienten im ÖPV kritisch bzw. für 49 % (IV) bzw. 75 % (ÖPV) nicht realistisch (Tab. 3; Abb. 3). Die verfügbare Verbindung im ÖPV wurde in 72 % der Fälle als gut bewertet und in 28 % der Fälle als schlecht. Für fast ein Viertel (*n* = 239) der Patienten lag zur nächstgelegenen TK keine ÖPV-Verbindung vor. Die Erreichbarkeit war aufgrund der bei diesem Versorgungsangebot größten regionalen Verteilungsunterschiede weniger durch Wohnortcharakteristika als durch die Angebotsseite geprägt (Tab. 3; Abb. 3).

**Tab. 3:** Erreichbarkeit der jeweils nächstgelegenen universitären Schmerzambulanz und des nächstgelegenen (teil-)stationären Angebots zur Interdisziplinären Multimodalen Schmerztherapie (*IMST*), dargestellt für Individualverkehr und öffentlichen Personenverkehr (*ÖPV*)

Bundesland	Universitäre Schmerzambulanz									Teilstationäre IMST									Stationäre IMST								
	*Fahrzeit IV*				*Fahrzeit ÖPV*					*Fahrzeit IV*				*Fahrzeit ÖPV*					*Fahrzeit IV*				*Fahrzeit ÖPV*				
	*Cut-off* *30* *min*		*Cut-off* *45* *min*		*Cut-off* *45* *min*		*Cut-off* *60* *min*		*Anteil ohne Verbindung*	*Cut-off* *30* *min*		*Cut-off* *45* *min*		*Cut-off* *45* *min*		*Cut-off* *60* *min*		*Anteil ohne Verbindung*	*Cut-off* *30* *min*		*Cut-off* *45* *min*		*Cut-off* *45* *min*		*Cut-off* *60* *min*		*Anteil ohne Verbindung*
	*% der verfügbaren Verbindungen*				*% der verfügbaren Verbindungen*				*% von Gesamtzahl*	*% der verfügbaren Verbindungen*				*% der verfügbaren Verbindungen*				*% von Gesamtzahl*	*% der verfügbaren Verbindungen*				*% der verfügbaren Verbindungen*				*% von Gesamtzahl*
	*≤* *30*	*>* *30*	*≤* *45*	*>* *45*	*≤* *45*	*>* *45*	*≤* *60*	*>* *60*		*≤* *30*	*>* *30*	*≤* *45*	*>* *45*	*≤* *45*	*>* *45*	*≤* *60*	*>* *60*		*≤* *30*	*>* *30*	*≤* *45*	*>* *45*	*≤* *45*	*>* *45*	*≤* *60*	*>* *60*	
Deutschland *n* = 1000	30	70	51	49	20	80	32	68	24	32	68	51	49	17	83	25	75	24	61	39	86	14	39	61	52	48	24
Baden-Württemberg (BW) *n* = 134	16	84	28	72	7	93	11	88,9	46	10	90	21	79	8	92	11	89	51	44	56	81	19	23	77	39	61	43
Bayern (BY) *n* = 158	27	73	50	51	22	78	37	63	44	66	34	90	10	34	66	52	48	46	51	49	78	22	25	75	34	66	48
Berlin (BE) *n* = 44	84	16	100	0	79	21	89	11	0	93	7	100	0	75	25	93	7	0	98	2	100	0	77	23	98	2	0
Brandenburg (BB) *n* = 30	3	97	10	90	0	100	10	90	3	20	80	57	43	11	89	22	78	10	47	53	77	23	41	59	41	59	10
Bremen (HB) *n* =8	12	88	75	25	0	100,0	0	100	12	100	0	100	0	0	100	0	100	0	87	13	87	13	100	0	100	0	13
Hamburg (HH) *n* = 22	68	32	95	5	45	55	64	36	0	95	5	100	0	82	18	91	9	0	95	5	100	0	82	18	91	9	0
Hessen (HE) *n* = 76	24	76	59	41	8	92	29	71	33	20	80	43	57	9	91	16	84	16	54	46	83	17	31	69	42	58	18
Mecklenburg-Vorpommern (MV) *n* = 19	26	74	47	53	20	80,0	20	80	47	47	53	74	26	20	80	50	50	47	5	95	26	74	0	100	0	100	53
Niedersachsen (NI) *n* = 96	10	90	22	78	7	93	8	92	8	5	95	11	89	2	98	4	96	13	58	42	87	13	21	79	38	62	10
Nordrhein-Westfalen (NW) *n* = 216	56	44	81	19	28	72	47	53	4	18	82	35	65	3	97	11	89	0	80	20	97	3	53	47	70	30	1
Rheinland-Pfalz (RP) *n* = 49	4	96	35	65	8	92	25	75	75	51	49	90	10	50	50	67	33	75	53	47	75	25	29	71	57	43	71
Saarland (SL) *n* = 12	25	75	83	17	8	92	17	83	0	25	75	75	25	8	92	8	92	0	83	17	92	8	30	70	60	40	17
Sachsen (SN) *n* = 49	20	80	22	78	18	82	24	76	8	47	53	75	25	29	71	38	62	8	67	33	84	16	49	51	51	49	25
Sachsen-Anhalt (ST) *n* = 26	23	77	50	50	6	94	6	94	38	100	0	23	77	0	100	0	100	61	61	39	92	8	25	75	42	58	54
Schleswig-Holstein (SH) *n* = 35	23	77	49	51	6	94	13	87	11	14	86	31	69	6	94	18	82	6	54	46	97	3	15	85	21	79	3
Thüringen (TH) *n* = 26	8	92	35	65	0	100	0	100	8	19	81	50	50	0	100	4	96	8	54	46	77	23	17	83	25	75	8

### Stationäre IMST

Insgesamt wurden 388 Einrichtungen identifiziert, welche die OPS 8‑918 im Bezugsjahr abgerechnet haben (durchschnittlich 174,28 ± 216,90 Fälle/Jahr). Die zugehörigen Krankenhäuser hatten eine Gesamtbettenzahl von 375,27 ± 355,90 Betten (min. 13, max. 3011, je 35 % freigemeinnützige und öffentliche und 30 % private Träger). Davon wiesen 259 Einrichtungen mehr als 75 Fälle/Jahr der OPS 8‑918 auf (242,64 ± 237,13). Letztere waren mehrheitlich unter freigemeinnütziger Trägerschaft (38 %, privat 33 %, öffentlich 29 %) und wiesen eine durchschnittlich niedrigere Gesamtbettenzahl auf als die Gesamtheit aller einbezogenen Krankenhäuser (328,31 ± 310,35, min. 27, max. 3011). Stationäre IMST-Angebote gehörten mehrheitlich zu anästhesiologischen Abteilungen (39 %) oder waren eigenständige Schmerzkliniken (38 %, andere: 12 % Orthopädie-Unfallchirurgie, 5 % Neurologie, 2 % Innere Medizin, 4 % sonstige) und wurden fachärztlich in 67 % anästhesiologisch geführt (14 % orthopädisch-unfallchirurgisch, 8 % neurologisch, 3 % internistisch, 8 % sonstige).

Die nächste stationäre IMST war durchschnittlich 25,83 ± 22,46 km vom Wohnort entfernt (min. 0,5, max. 244 km, Tab. 1), was Fahrkosten/einfache Strecke von 7,74 ± 6,74 € entsprach (min. 0,15, max. 73,2 €). Bzgl. der Fahrzeit war dies für einen Großteil der Patienten grundsätzlich realistisch mit dem Auto zu erreichen (Tab. 3; Abb. 3). Die ÖPV-Verbindung wurde in 85 % der Fälle als gut und für 15 % schlecht eingestuft. Für 237 Patienten lag keine ÖPV-Verbindung vor. Die Fahrzeit im ÖPV lag bei ca. der Hälfte der Modellpatienten über der kritischen Schwelle (Tab. 3; Abb. 3).

## Diskussion

Die Ergebnisse zeigen, dass bundesweit relevante Unterschiede je nach Wohnort in der Erreichbarkeit von Einrichtungen zur speziellen Schmerzbehandlung bestehen. Für ländliche Wohnorte lagen erwartungsgemäß die längsten Anfahrtszeiten vor, wobei die Unterschiede nicht zwingend abhängig von der Wohnortcharakteristik sind, sondern auch von dem regionalen, meist bundeslandspezifisch vorgehaltenen Versorgungsspektrum. Mit Blick auf die Behandlung eines chronischen Krankheitsbilds mit langfristiger therapeutischer Zielsetzung, mit der Notwendigkeit abgestufter Versorgung – längerfristig ambulant, intermittierend (teil-)stationär –, offenbaren die Ergebnisse aus Patientenperspektive eine teils kritische Versorgung. Zudem offenbart sich im Kontext der dynamischen und derzeitig kritischen ökonomischen Situation der Krankenhäuser eine Gefahr für bestehende IMST-Strukturen, da deren Anteil im stationären Bereich stark von freigemeinnützigen Krankenhäusern getragen wird, die derzeit im Kontext drohender Krankenhausschließungen besonders gefährdet sind [8, 18].

### Ambulante Versorgung

Von den meisten Wohnorten aus konnten spezialisierte ambulante Einrichtungen und meist auch universitäre Schmerzambulanzen realistisch erreicht werden. Dennoch muss unterschieden werden, ob es sich um einzelne ambulante Kontakte handelt oder ob es einer engeren therapeutischen Begleitung und Anbindung bedarf, ggf. mit regelmäßig notwendigem Erscheinen in der Einrichtung oder der Frage von Ambulantisierung stationärer Leistungen – auch unabhängig von der Frage finanzieller und qualitativer Umsetzbarkeit. Die Untersuchung erlaubt keine Aussagen zu Versorgungskapazitäten, Anzahl der jeweiligen Behandler und entsprechender Sitze. Die in der Analyse operationalisierten drei ambulanten Einrichtungsgruppen sind nur selten parallel bestehende Strukturen, sondern überschneiden sich. Die Erreichbarkeit relativiert sich zudem, wenn Einrichtungskapazitäten erschöpft sind. Die geltende Höchstgrenze für EBM 30702 und 30704 sind 300 Fälle/Quartal. Regelhaft wird diese durch die Kassenärztlichen Vereinigungen hochgesetzt (KV, in Niedersachsen beispielsweise auf 450 Fälle/Quartal), was schnell die Grenze der Plausibilität erreichen lässt, sodass Praxen diese entweder nicht voll ausschöpfen oder dies nur unter Vernachlässigung der Behandlungsintensität funktioniert. Stand 2016 nahmen 1206 Ärzte über die QSV teil, was rechnerisch – hochgesetzte Fallzahlen vorausgesetzt – nur ca. einer halben Million Menschen in Deutschland eine Versorgung ermöglichen würde [29]. Das Versorgungsdefizit wird deutlich, bedenkt man, dass von bis zu 6 Mio. Behandlungsbedürftigen ausgegangen wird [20]. Hilfreich könnte diesbezüglich die ergänzende Nutzung von telemedizinischen Angeboten sein, um die Betroffenen zu entlasten. Das eignet sich jedoch nicht für alle Patienten.

Ein weiterer kritischer Punkt ist, dass mit dem baldigen altersbedingten Ausscheiden vieler QSV-Schmerztherapeuten zu rechnen ist [29]. Stand 2018 gaben 58 % an, innerhalb der nächsten 10 Jahre ihre Tätigkeit einzustellen [24, 29]. Besonders kritisch: Von diesen sahen zuletzt ca. 50 % eine Nachfolge bisher nicht gesichert [24]. Dass in den meisten KV-Bereichen die Schmerzmedizin keine eigene Planungskategorie darstellt, erschwert es, ambulante schmerzmedizinische Angebote bei Nachfolgen zu sichern. Freiwerdende Sitze können innerhalb der Fachgruppe auch an nicht schmerzmedizinisch tätige Ärzte gehen. Hilfreiche Maßnahmen wären zum einen die Förderung von Weiterbildungsstellen, analog z. B. zur Allgemeinmedizin, oder die spezifische Ausschreibung von Sitzen zur Schmerztherapie. Ein effektiver Schritt wäre, bei der Vergabe sicherzustellen, dass die Weitergabe von Sitzen schmerztherapeutischer Praxen ausschließlich an Schmerztherapeuten erfolgt, wie beispielsweise von der KV Schleswig-Holstein 2016 erstmalig eingeführt [31].

### (Teil-)stationäre Behandlung

Für einzelne Angebote, wie die stationäre Schmerztherapie, wird teils eine regionale Überversorgung diskutiert. Diese entsteht primär im rechnerischen Vergleich von Behandlungsleistungen und regionaler Bevölkerung. Doch gerade im stationären Sektor kommt es in der Realität teils zu regionsüberschreitender Versorgung [26]. Gerade bzgl. der möglichen Therapie in einer Schmerztagesklinik lagen die Bundesländer besonders weit auseinander – der Faktor der Regionalcharakteristika machte sich in den Bundesländern unterschiedlich bemerkbar, zeigte aber, dass auch in Flächenländern mit großen ländlichen Regionen, wie in Bayern oder Mecklenburg-Vorpommern, durchaus tagesklinische Therapiekonzepte auch für einen relativ großen Anteil der Patienten umsetzbar sein könnten. Ein im Vergleich regional breit verteiltes tagesklinisches Angebot wiesen nur Bayern, Rheinland-Pfalz und Sachsen bzw. für ihren Bereich die Stadtstaaten Hamburg und Berlin auf, teils Mecklenburg-Vorpommern und Brandenburg. Nordrhein-Westfalen hatte TK beispielsweise nur im Nordosten (weitgehend im Zuständigkeitsbereich der Ärztekammer Ostwestfalen-Lippe). In Niedersachsen existiert eine TK. Für die Mehrheit der Menschen in Deutschland ist somit eine tagesklinische Schmerzbehandlung, insb. bei täglicher Anreise, kein realistisch verfügbares und erreichbares Behandlungsangebot. Die vollstationäre IMST, die im Vergleich zur teilstationären im letzten Jahrzehnt eine enorme Verbreitung erfahren hat, ist, wie die Ergebnisse dieser Studie zeigen, in den meisten Fällen die einzige realistisch erreichbare Option zur IMST. Stationäre Behandlung kann jederzeit angeboten werden und obliegt nicht der Genehmigung; die Eröffnung einer teilstationären Option wird in vielen Bundesländern bzw. Kammerbereichen ausdrücklich nicht oder nur zurückhaltend unterstützt.

Das konterkariert nicht nur den Ansatz abgestufter Versorgungskonzepte, sondern macht deutlich, dass für viel Menschen in Deutschland mit chronischen Schmerzen stationäre IMST-Konzepte die einzig realistisch umsetzbaren IMST-Angebote darstellen [7, 28]. Zu „gesunden“ Patienten bleiben damit oft nur monomodale und beim chronischen Schmerz meist als nicht wirksam eingestufte ambulante und nicht verzahnte Therapieangebote. Denn im Kontext der Indikationsprüfungen durch die Leistungsträger für stationäre Behandlungen steht weniger das Kriterium der „chronischen Schmerzerkrankung an sich“ im Fokus, sondern mehr die Frage nach der Notwendigkeit des Einsatzes der besonderen Mittel eines Krankenhauses [6]. Die fehlende Erreichbarkeit/Verfügbarkeit lokoregionaler Angebote rechtfertigt die Indikation zur stationären Aufnahme nicht. Außerhalb von Forschungsprojekten wie PAIN 2.0 existieren bisher im Sinne der Definition enger und systematisch abgestimmter Zusammenarbeit zwischen den verschiedenen beteiligten Fachdisziplinen (z. B. in Form systematischer Teambesprechungen) und untereinander fortlaufend abgestimmter räumlicher und inhaltlicher Verknüpfung formal keine interdisziplinären multimodalen Therapieprogramme im ambulanten Bereich mit vergleichbarer Therapieintensität [28, 33]. Das kann zum Paradoxon führen, dass Patienten erst stärker chronifizieren oder weiter eine nicht effektive monomodale ambulante Therapie erhalten müssen, bis dann im Verlauf die Indikation für den Einsatz der Mittel des Krankenhauses begründbar ist, oder aber Symptome und Therapie aggraviert werden müssen, um die adäquate Versorgung zu ermöglichen. Hilfreich ist das in einem verhaltensmedizinisch ausgerichteten Behandlungsansatz nicht.

Zudem bestehen konkrete Pläne der aktuellen Regierungskoalition, die Sektorentrennung teils zu überwinden und Ambulantisierung zu fördern. Zu schaffende Hybrid-DRG, deren Entwicklung geplant ist, sollen ermöglichen, dass bestimmte Leistungen sowohl von ambulanten Anbietern als auch von Krankenhäusern zu gleichen Konditionen erbracht werden können [32]. Konkret ist die Schmerzmedizin ein hier genannter Versorgungsbereich [12]. Doch würde die Ambulantisierung (unabhängig von der qualitativen Umsetzbarkeit) komplexer interdisziplinärer Behandlungskonzepte bei den komplex erkrankten Patienten, deren Behandlung oft eine enge interdisziplinäre Vernetzung und Verfügbarkeit auch über die Schmerzmedizin hinaus erfordert, ersetzen? Lediglich die regional bessere Verfügbarkeit ambulanter Einrichtungen wäre, wie unsere Analyse zeigt, ein Aspekt, der dafürspräche. Paradox erscheint, dass in vielen Bereichen aus Qualitätsgründen gerade auf Mindestmengen und enge Strukturkriterien gesetzt wird [22]. Ausgerechnet für die Schmerzmedizin wird in die gegenteilige Richtung (zumindest) nachgedacht [12]. Heterogenität in der Behandlungsroutine drückt sich auch in den Fallzahlen unserer Analyse und den zahlreichen IMST-Anbietern mit sehr niedriger Fallzahl aus. Im Sinne hoher Behandlungsqualität wären eher Überlegungen zielführender, wie es gelänge, auch ohne primär vollstationäre Indikation Menschen zur Therapie an hochspezialisierten Zentren zusammenzuführen. Die im Vergleich zu unserer Analyse flächendeckendere Erreichbarkeit teilstationärer Einrichtungen wäre ein weiterer wichtiger Schritt in Richtung einer zielgerichteteren Therapie- und Ressourcenallokation.

### Zumutbarkeitsgrenzen und Sicherstellung der Versorgung

Die von uns für die Analyse gesetzten Grenzwerte für realistische bzw. kritische Erreichbarkeit schmerzmedizinischer Einrichtungen entsprechen in etwa denen der KBV bzgl. zumutbarer Fahrzeit und Entfernung zum Facharzt (siehe Methodenteil). Diese Zumutbarkeitsgrenzen haben beispielsweise bei der Terminvermittlung durch die KVen Konsequenzen. Bei Überschreitung hat die Terminservicestelle einen Behandlungstermin in einem geeigneten Krankenhaus zu vermitteln (siehe auch Sozialgesetzbuch [SGB] Fünftes Buch [V] – § 75). Im Übrigen werden ähnliche Werte auch bei der Frage der Sonderbedarfszulassung über die KVen herangezogen [21]. Eine Sicherstellung eines Versorgungsangebots in der nächsthöheren Versorgungsstufe bei fehlenden Alternativen ist im (teil-)stationären Sektor keine Option. Da in der Versorgungsrealität kein ambulantes IMST-Programm in der Regelversorgung besteht oder, wie unsere Ergebnisse darstellen, für Menschen der meisten Wohnorte in Deutschland eine teilstationäre IMST nicht in realistischer Erreichbarkeit für tägliche An- und Abfahrten liegt, bleibt den Betroffenen formal eine IMST verwehrt. Bei den Angeboten über die Terminvergabe der KVen sind ausdrücklich örtliche Verhältnisse sowie die öffentliche Verkehrsanbindung zu berücksichtigen [5, 21]. Egal ob aufgrund von patientenseitigen Faktoren oder struktureller Unterversorgung im (teil-)stationären Sektor zählt bei Prüfungen des Medizinischen Dienstes weder der Aspekt der Nichterreichbarkeit noch der Aspekt der Zumutbarkeit der Anfahrtszeit und Strecke. Hier ist es auch unerheblich, ob es Alternativen in der Realität gibt.

## Limitation

Die Analyse basiert je nach Versorgungsstruktur auf unterschiedlichen Datengrundlagen. Für die ambulante QSV-Versorgung erfolgte die Identifizierung mangels anderer Übersichten anhand von Behandlungskontakten von Patienten einer großen deutschen Krankenversicherung. In Abhängigkeit regionaler Unterschiede in der Versichertenstruktur könnte es sein, dass regional einzelne Einrichtungen nicht erfasst worden sind, obwohl wir davon ausgehen, dass in jeder Einrichtung mindestens ein Kontakt im Bezugszeitraum eines entsprechend versicherten Mitglieds erfolgte. Ähnliches gilt für die teilstationäre Schmerztherapie. Die vollstationären Angebote wurden anhand des Deutschen Krankenhausverzeichnisses erfasst, dessen Datengrundlage die Qualitätsberichte der Einrichtungen sind. Hier vermögen Unsicherheiten der Analyse aufgrund von Fehl- oder Nichtangaben der Einrichtungen in den Qualitätsberichten bestehen.

Die dargestellten Ergebnisse zu QSV-Einrichtungen legen nahe, dass 22 Einrichtungen einbezogen wurden, die zwar EBM 30700, aber nie EBM 30702 abrechnen. Erstere ist eine „Grundpauschale“ und 30702 eine Zusatzpauschale. Die Abrechnung von EBM 30700 ohne EBM 30702 ist zwar in Einzelfällen möglich, aber nur in einer Praxis, die grundsätzlich auch die Leistung gemäß Ziffer 30702 erbringt (z. B. bei einem Patienten mit persönlichem Arzt-Patienten-Kontakt ohne weitere Abklärung oder Untersuchung bei einem Wiederholungsrezept). Aufgrund der zu erwartenden hohen Überlappung der EBM-Gruppen (z. B. rechnen „30704er“-Einrichtungen i. d. R. auch die anderen beiden EBM-Ziffern ab) darf dies nicht als per se unterschiedliche Kohorten angesehen werden, sondern stellt mit der Differenzierung von EBM 30702 und 30704 lediglich eine Operationalisierung für die Darstellung unterschiedlicher Schwerpunktanteile und Struktur (nachzuweisende Vernetzungsstruktur) dar. Die Analysegruppen EBM 30700 und EBM 30702 enthalten somit auch „EBM-30704-Einrichtungen“.

Die Fahrzeitanalyse erfolgte zur bestmöglichen Reduktion eines Bias unter standardisierten und zeitlich definierten Kriterien. Dennoch unterliegen die Ergebnisse damit programminternen Algorithmen, die für uns als Nutzer nicht nachzuvollziehen sind. In der Vorbereitung der Untersuchung testeten wir verschiedene frei verfügbare Routenplaner, unter denen sich das genutzte Programm am praktikabelsten und realistischsten – insbesondere aufgrund der dargestellten Ist-Zeiten – darstellte. Eine relevante Limitation bestand bei den Recherchen darin, dass nicht alle Verkehrsverbünde ihre Fahrpläne mit Google Maps© abgleichen. In diesen Bereichen bestand somit bundeslandabhängig ein Bias, wenn es darum ging, die Fahrzeit im ÖPV darzustellen. Dies beeinflusste sicherlich, ohne dass dies in der Recherche abschließend nachvollziehbar war, einige der Fälle, bei denen keine oder nur schlechte Verbindungen ausfindig gemacht werden konnten.

## Fazit für die Praxis

Aus Patientenperspektive verdeutlichen die Ergebnisse dieser Modellanalyse eine teils kritische Situation in Deutschland bzgl. abgestufter schmerzmedizinischer Versorgung. Für viele Menschen mit chronischen Schmerzen, deren Mobilität und Belastbarkeit oft stark eingeschränkt ist, ist die Erreichbarkeit je nach Versorgungsform unrealistisch und nicht zumutbar. Versorgungskonzepte und gesundheitspolitische Planungen sollten die spezifischen Erfordernisse in der abgestuften Behandlung von Menschen mit chronischen Schmerzen auch mit Blick auf Erreichbarkeit und damit verbundene Umsetzbarkeit konzeptionell berücksichtigen.

## Supplementary Information

Grafische Darstellung der Verteilung der randomisiert generierten Wohnorte der 1000 analysierten Modellpatienten in Deutschland

## Data Availability

Aufgrund von Datenschutzbestimmungen sind die Daten nur teilweise verfügbar. Der Zugang zu diesen Informationen ist nur unter angemessenen Bedingungen und auf berechtigten Grundlagen möglich. Auch konnten wir aus Datenschutzgründen nicht alle Versorgungseinrichtungen grafisch in Karten darstellen.
