# Supplementary figures and images for: Regional comparison of specialized outpatient and (partial) inpatient pain medicine care in Germany
Source: Schmerz. 2024 Sep 18;40(1):16–30. [Article in German] doi: 10.1007/s00482-024-00829-7 (PMC12858620; doi:10.1007/s00482-024-00829-7)

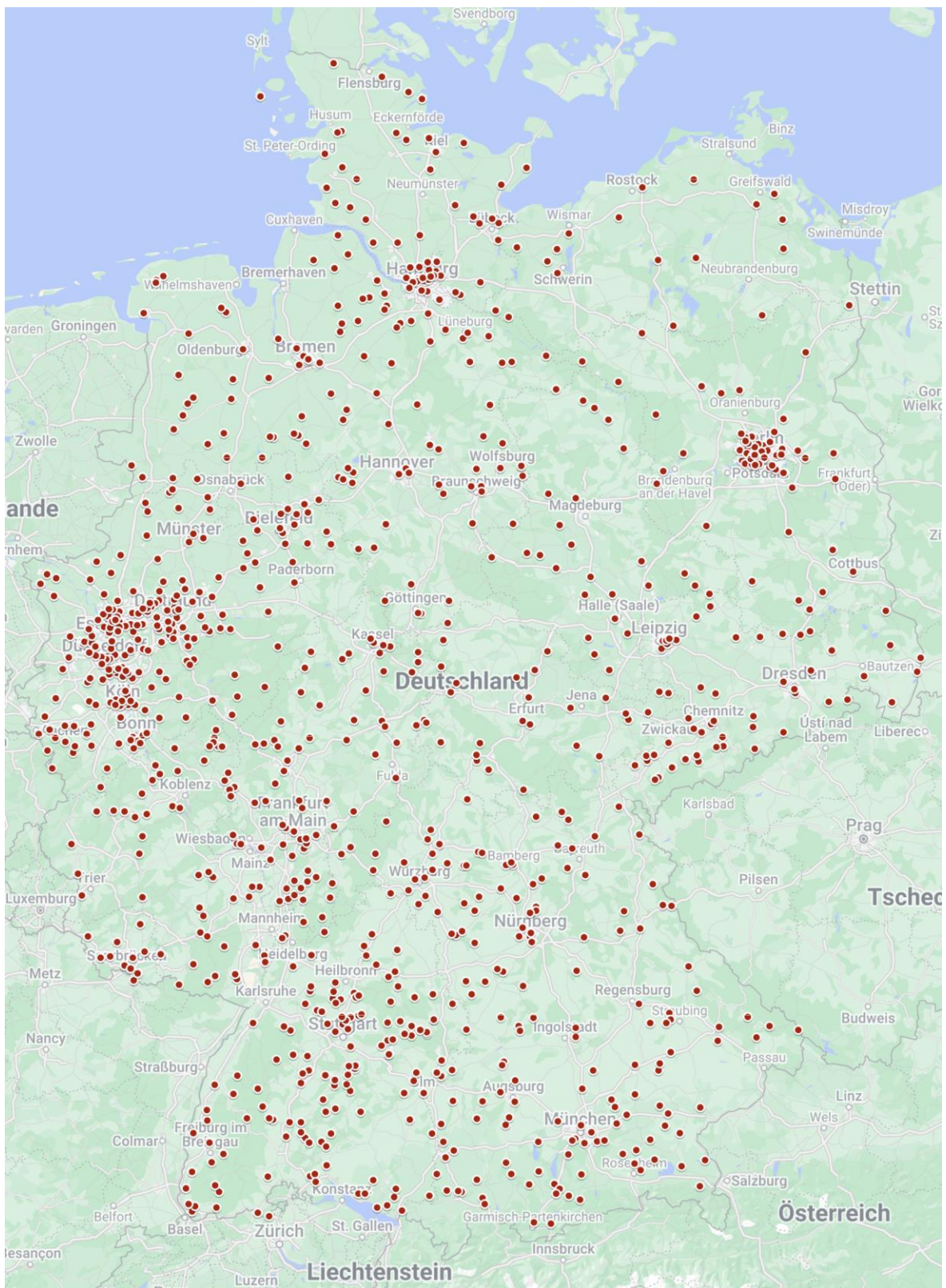

Erstellt mit: Kartendaten © 2024 GeoBasis-DE/BKG (2009 ©), Google, Inst. Geogr. Nacional

Supplement: Supplementary file 1 — Grafische Darstellung der Verteilung der randomisiert generierten Wohnorte der 1000 analysierten Modellpatienten in Deutschland [file 482_2024_829_MOESM1_ESM.pdf]
